# Predation by Red Foxes (*Vulpes vulpes*) at an Outdoor Piggery

**DOI:** 10.3390/ani6100060

**Published:** 2016-10-08

**Authors:** Patricia A. Fleming, Shannon J. Dundas, Yvonne Y. W. Lau, John R. Pluske

**Affiliations:** School of Veterinary and Life Sciences, Murdoch University, Murdoch, WA 6150, Australia; shannon.dundas@dpi.nsw.gov.au (S.J.D.); yvonne_lauyiwen@hotmail.com (Y.Y.W.L.); j.pluske@murdoch.edu.au (J.R.P.)

**Keywords:** behaviour, free-range, outdoor, piggery, piglet, predation

## Abstract

**Simple Summary:**

Predation of piglets by red foxes is a significant risk for outdoor/free-range pork producers, but is often difficult to quantify. Using remote sensing cameras, we recorded substantial evidence of red foxes taking piglets from around farrowing huts, and found that piglets were most likely to be recorded as “missing” over their first week. These data suggest that fox predation contributed to the marked production differences between this outdoor farm and a similar-sized intensive farm under the same management, and warrant greater control of this introduced, invasive predator.

**Abstract:**

Outdoor pig operations are an alternative to intensive systems of raising pigs; however for the majority of outdoor pork producers, issues of biosecurity and predation control require significant management and (or) capital investment. Identifying and quantifying predation risk in outdoor pork operations has rarely been done, but such data would be informative for these producers as part of their financial and logistical planning. We quantified potential impact of fox predation on piglets bred on an outdoor pork operation in south-western Australia. We used remote sensor cameras at select sites across the farm as well as above farrowing huts to record interactions between predators and pigs (sows and piglets). We also identified animal losses from breeding records, calculating weaning rate as a proportion of piglets born. Although only few piglets were recorded lost to fox predation (recorded by piggery staff as carcasses that are “chewed”), it is likely that foxes were contributing substantially to the 20% of piglets that were reported “missing”. Both sets of cameras recorded a high incidence of fox activity; foxes appeared on camera soon after staff left for the day, were observed tracking and taking live piglets (despite the presence of sows), and removed dead carcasses from in front of the cameras. Newly born and younger piglets appeared to be the most vulnerable, especially when they are born out in the paddock, but older piglets were also lost. A significant (*p* = 0.001) effect of individual sow identification on the weaning rate, but no effect of sow age (parity), suggests that individual sow behavior towards predators influences predation risk for litters. We tracked the movement of piglet carcasses by foxes, and confirmed that foxes make use of patches of native vegetation for cover, although there was no effect of paddock, distance to vegetation, or position on the farm on weaning rate. Trials with non-toxic baits reveal high levels of non-target bait interference. Other management options are recommended, including removing hay from the paddocks to reduce the risks of sows farrowing in open paddocks, and covering or predator-proof fencing the pig carcass pit. Results of this study will have increasing relevance for the expanding outdoor/free-range pork industry, contributing to best practice guidelines for predator control.

## 1. Introduction

As consumer demands have a growing influence over how production animals are reared and housed [1,2], livestock producers are increasingly exploring more natural, outdoor housing. In outdoor piggeries, the sows have free movement in group paddocks, with wallows and open deep-litter shelters or basic huts where they will farrow. In addition to potential vulnerability to lay-overs and crushing [3,4], piglets born under these natural conditions are also exposed to a range of biosecurity and predation risks.

Biosecurity is a concern for outdoor piggeries [5] due to the risk of contamination of herds through increased exposure to wildlife, especially feral pigs (*Sus scrofa*), e.g., [6,7,8]. Another major issue is around the predation of piglets [9], although there is a paucity of published data that identifies the extent of piglet losses for commercial outdoor farms [4,10,11,12]. Young piglets that are reared outside may be submitted to predation by red foxes (*Vulpes vulpes*), corvids (including ravens, rooks, and crows), or mustelids (including badger species) [4].

In the Australian pork industry, approximately 11% of sows are kept and have litters outdoors, with approximately 40% of these being located in Western Australia [13]. Although outdoor production represents only a small proportion of the Australian pork industry, this production system is generating increasing interest, producing a top quality, niche market product that mainly caters for the high end retail and restaurant trades [14]. For the majority of outdoor pork producers, issues of biosecurity and predation control require significant management and (or) capital investment, which can limit growth of the industry. Identifying and quantifying predation risk in outdoor pork operations has rarely been done, but such data would be informative for these producers as part of their financial and logistical planning.

In collaboration with a commercial outdoor pork operation, we quantified the potential impact of predation and investigated potential management options to mitigate this issue. The objectives of this study were to:

- Identify the level of threat posed by fox predation using infra-red camera trap monitoring to determine the level of fox presence in and around the outdoor piggery, and test whether there was a temporal and spatial overlap between piglets and foxes.
- Use piggery records of births and weaning to identify whether piglets were more vulnerable to fox predation at a particular age, whether predation risk increased with increased proximity to native vegetation, or between first time (gilts) and experienced sows.
- Carry out a preliminary study to identify factors that would influence the efficiency of control baiting.

## 2. Methods

We worked at a commercial Western Australian outdoors pork operation. This operation has approximately 3500 sows and weans an average of 1000 piglets per week. Each farrowing paddock (each 400 m^2^) has nine sows. The sows were introduced to the paddock a few days before their expected farrowing date, and each sow selected one of the nine huts that were available. Fenders (~30 cm tall) were placed at each hut entrance to keep piglets in the hut until ~7–14 days of age, after which the fenders were removed and the piglets were allowed to interact with other piglets and sows in the paddock until they were weaned from the paddocks at 21–28 days of age. The sows were fed from tall feeders, but piglets had access to spilled food on the ground as well as the freedom to move in and out the farrowing paddock, and therefore had access to adjacent paddocks as well as adjacent vegetation around these paddocks.

### 2.1. Identify the Level of Threat Posed by Fox Predation

Eight Reconyx HC500 remote infrared sensor cameras (RECONYX Inc., Holmen, WI, USA; set on “Rapidfire” to take five photos per trigger) were secured to a tree or fence post at a height of 0.5 m facing onto roads at key monitoring sites: between an open field and farrowing paddocks (*n* = 2 cameras), adjacent to patches of native vegetation amongst the farrowing paddocks (*n* = 4 cameras), around the property perimeter at the border with adjacent blue gum (*Eucalyptus globulus*) plantations (*n* = 1 camera), and on the access road to the farm carcass pit (*n* = 1 camera). These monitoring site cameras were left in situ for a period of 112 days (August–December 2014).

An additional 11 cameras were mounted to the front of 11 farrowing huts spread across nine farrowing paddocks that were selected based on the presence of sows that were about to farrow or had farrowed within days of commencing the study. These cameras were left in place for 1 month (November–December 2014) and recorded from around birth to weaning for the litters using each hut.

Each “capture event” was counted as a fox seen on camera, with a minimum interval of 15 min between consecutive events, noting that this interval varies between studies [15]. We compared the activity patterns (numbers of records per hour interval) for the cameras placed out at the long-term monitoring sites, those on the farrowing huts, and for cameras placed on non-toxic baits around the perimeter of the farm (see Section 2.3 below). For piglets, we recorded the numbers of photograph records, because we could not identify individual “capture events”.

### 2.2. Farrowing and Weaning Records—Factors that Increase Risk of Fox Predation

We analyzed the farrowing data from the previous 3 years (2012–2014) for this operation. The number of piglets was recorded for each sow daily, allowing estimation of when piglets disappeared (these were recorded by staff as “missing” animals), deaths due to lay-overs, or those that were obviously due to predators (carcasses chewed). For every farrowing event, we identified the number of piglets born, the number weaned, and calculated the piglet’s age when a mortality event was recorded. The weaning rate (proportion of live piglets at farrowing that were successfully weaned) was calculated for each litter of each sow. The weaning rate (dependent variable) was analyzed using a mixed-model ANOVA with sow identification (ID) and farrowing paddock ID included as random factors, position on farm (edge of vegetation/open paddock or in the middle of pig paddocks) included as a fixed factor, and parity number and distance of farrowing paddock to closest native vegetation (determined from analysis of aerial photographs) included as covariates.

### 2.3. Preliminary Trials to Test Bait Presentation and Placement

Presentation of baits: We placed four non-toxic dried kangaroo meat “baits” (each ~100 g fresh weight) in front of the eight monitoring site cameras. Each “bait” was presented in one of four ways: suspended 1 m from the ground on fishing line from a metal gantry, wrapped in kangaroo hide, buried, and laid on the soil surface.

Where to lay baits: To compare bait-take in open versus forested areas, 36 Reconyx HC500 cameras were used to monitor 18 buried and 18 surface-laid non-toxic meat “baits” (nine of each presentation in open and forest). These cameras were left in place for 1 night (time was limited due to constraints on access to the site).

Tracking predator movements: To investigate where foxes would take piglet carcasses (cache or den site), we attached GPS (GT-120 i-gotU USB GPS Logger, MobileAction Technology, Inc. Taipei, Taiwan; recording location every 1 min with the smart tracking mode enabled) and VHF (V2G 154B glue-on VHF core, Sirtrack, New Zealand) trackers to each of 32 piglet carcasses. We used steel cable ties running around the middle of the carcass to keep the GPS unit on the surface of the carcass. The carcasses were placed around the farm and monitored over 4 nights (2 nights in November and 2 nights in December 2014) using Reconyx HC500 cameras. Using the VHF signals, we then retrieved the GPS units and downloaded the data to identify where the carcasses had been carried.

## 3. Results

### 3.1. Identify the Level of Threat Posed by Fox Predation

**Figure 1:**
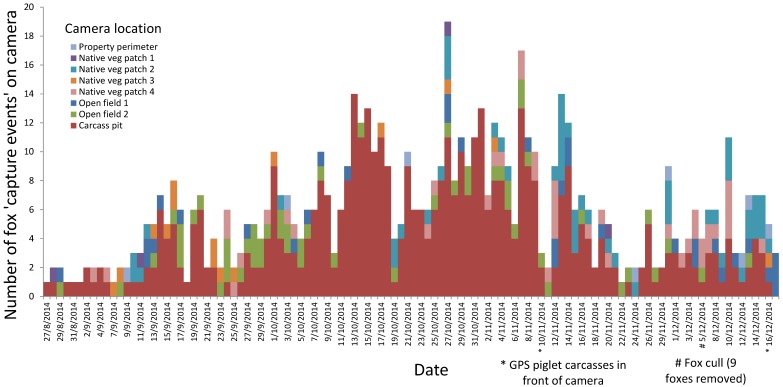
Fox activity at cameras placed in eight monitoring sites around the pork operation over 112 consecutive days (August–December 2014). Each fox capture event represents an individual fox seen on camera, with a minimum interval of 15 min between capture events.

Remote camera monitoring revealed a substantial level of fox activity across the long-term monitoring sites (Figure 1). We recorded an average of 6.0 ± 3.8 (max 19) fox capture events per night totaled over the eight long-term cameras left in place at the monitoring sites over 112 days. Activity of foxes was consistently high at the site next to the carcass pit (average 4.5 ± 3.4 fox capture events per night; max 14), with up to five foxes being captured in a single photo. Foxes were observed on a number of occasions returning from the carcass pit carrying scavenged piglet carcasses.

**Figure 2:**
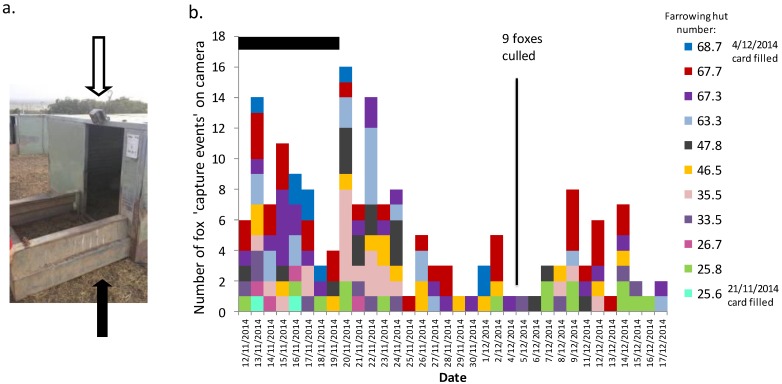
(**a**) Cameras mounted on 11 farrowing huts (white arrow shows camera; black arrow shows fenders that retain piglets within the huts for the first 10 days after they are born; (**b**) Numbers of capture events of foxes on farrowing hut cameras. The black bar above the graph indicates the time when fenders were in place. The numbers (key) identify the paddock and the hut within each paddock; two huts within paddock 67 and two within paddock 25 were monitored. The timing of a cull of nine foxes on the farm is indicated. Two cameras ran out of memory before the completion of the month—dates shown for these. The different colors reflect the individual huts monitored, and allow identification of where there are repeated visits to individual huts over consecutive nights.

**Figure 3:**
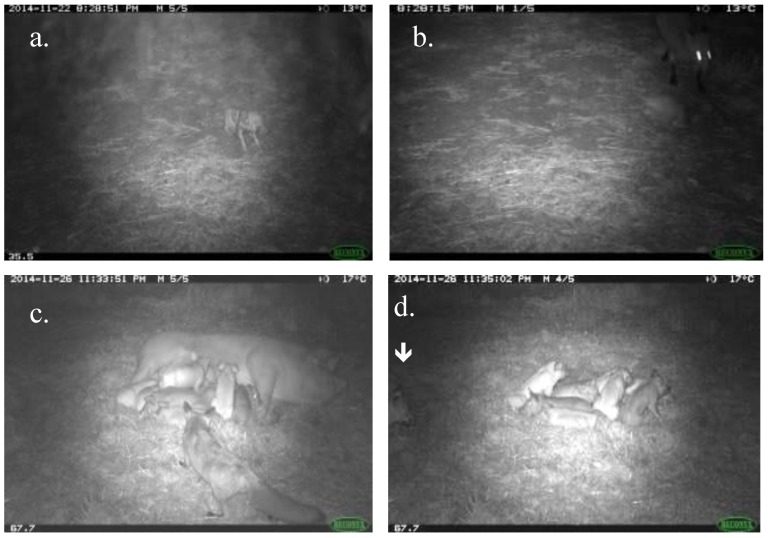
Photos of fox interaction with piglets. (**a**) A newborn piglet (umbilical cord and embryonic membrane still present) born outside of the farrowing hut and (**b**) fox taking the piglet; (**c**) fox taking a 2–3-week-old piglet from a sow feeding outside of a farrowing hut; (**d**) piglets showing limited wariness towards an approaching fox (arrow).

**Figure 4:**
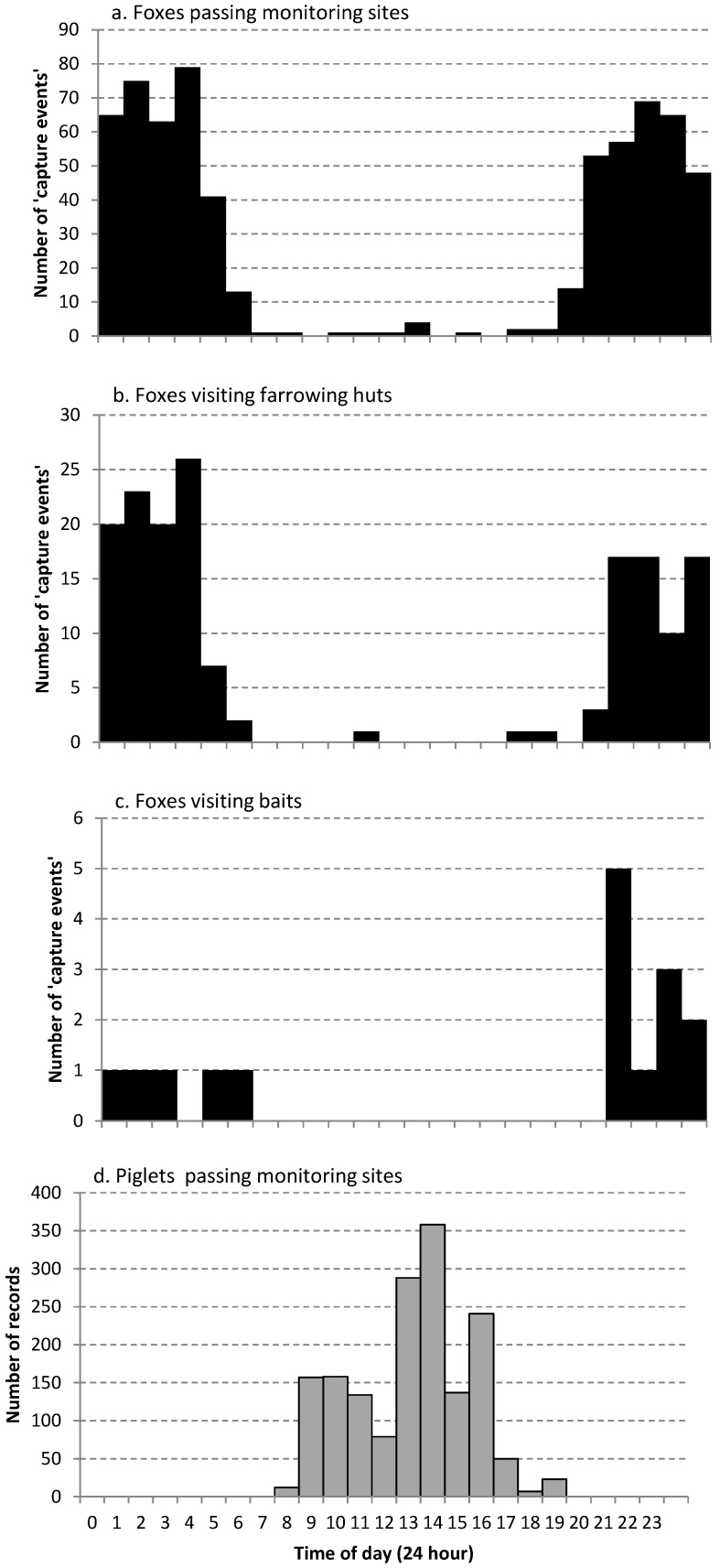
Temporal patterns in fox activity for cameras at three locations (**a**–**c**; total number of “capture events” **a**: *n* = 656; **b**: *n* = 165; **c**: *n* = 17) and for records of piglets passing cameras at the monitoring sites (**d**; *n* = 1683).

For cameras mounted at the farrowing hut entrances, we recorded an average of 15.0 ± 10.2 fox capture events per hut over the 1-month monitoring period (Figure 2). These cameras showed foxes approaching and even entering farrowing huts. We also recorded foxes stalking and taking live piglets in front of the huts where the piglets were either on their own (on the night that they were born), or older piglets as they were suckling from the sow lying outside the huts (Figure 3). We recorded an average of 7.8 ± 3.6 fox capture events per night (totalled over the 11 huts) over the first 8 days of monitoring, but then an increase to 10.4 ± 4.3 capture events per night for the 5 days immediately after the fenders were removed from the hut entrances (when piglets were no longer confined to within the huts). Fox activity at the farrowing huts subsequently declined after 2 weeks to 2.9 ± 2.1 capture events per night. Foxes became active on the farm almost immediately after staff finished work for the day (usually between 14:00–16:00 h). Foxes were observed on camera at the long-term monitoring sites (Figure 4a) and in front of farrowing huts (Figure 4b) during daylight hours. We noted more activity past bait sites (around the perimeter of the farm) for the early evening than in the morning hours (Figure 4c), but more fox activity in the early morning hours for our long-term monitoring sites (Figure 4a) and the farrowing huts (Figure 4b). Although piglets (Figure 4d) showed exclusively diurnal activity at the monitoring sites (which were all on roads and therefore outside the farrowing paddocks), the farrowing hut cameras provided evidence of foxes approaching the farrowing paddocks and interacting with piglets at night (Figure 3).

### 3.2. Farrowing and Weaning Records—Factors that Increase Risk of Fox Predation

**Figure 5:**
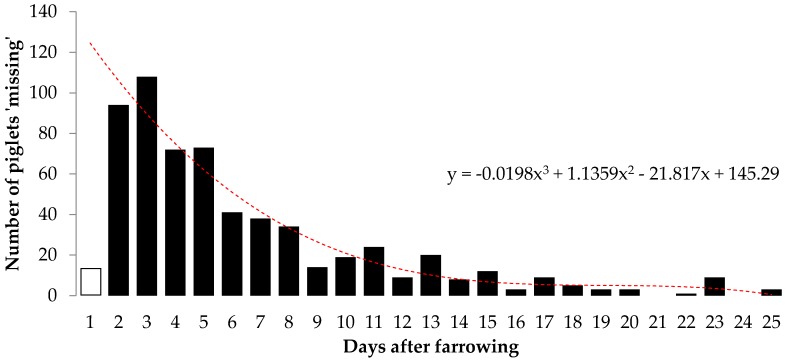
Distribution of records of ‘missing’ piglets by their age. Only 15 piglets were recorded as missing on their first day (white bar); however the red dotted line shows a polynomial line of best fit, which suggests that more piglets are likely lost on their first day (i.e., on the night they were born) than recorded.

In total, we recorded the weaning rates for 1953 farrowing events (individual litters per sow) on the farm. Sows gave birth to an average of 11.5 ± 3.6 piglets per litter (although we note that this calculation does not take into account losses of piglets on night 1, i.e., before they were recorded). Of 6143 piglet deaths recorded by the piggery staff, 75.6% were due to lay-overs and 3.9% due to low viability (e.g., failure to thrive, scours, splay legs, “shakes”, trauma/injury). Fox predation (carcass obviously chewed) was recorded in only eight instances (0.13%), while Australian ravens *Corvus coronoides* were recorded as the cause of death in 12 instances (0.20%). However, 20.1% of piglets at farrowing (recorded at the earliest occasion on the morning after the sows had farrowed) were recorded as “missing” or “unknown”. Piglets were most often recorded as “missing” during their first week (Figure 5), probably because they are light enough to be carried away by foxes. We assume that predators took the majority of these missing piglets, although the possibility of some human error also needs to be acknowledged.

**Figure 6:**
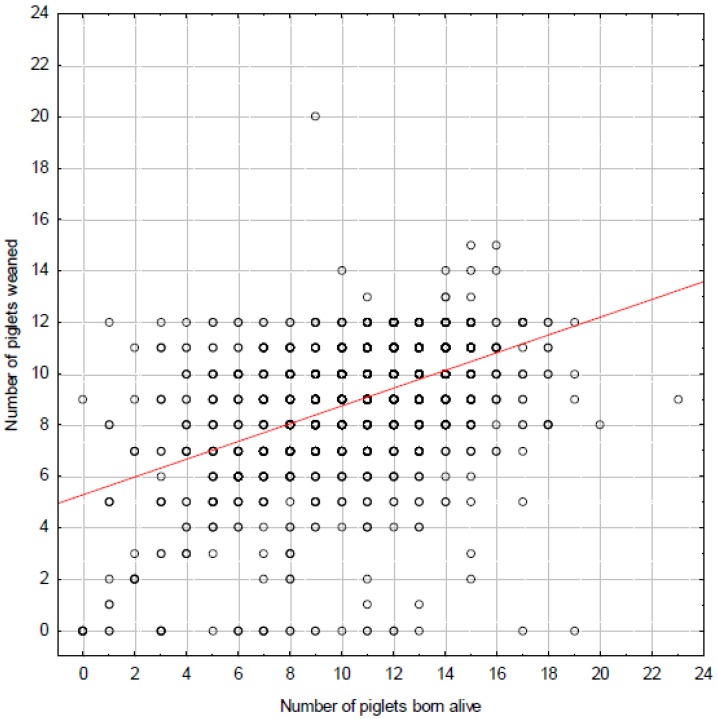
Relationship between the number of piglets born alive and weaned. Because piglets from sows with many young are fostered to sows with fewer piglets, the relationship between farrowed and weaned is not linear, i.e., the regression line (solid line) does not match linearity (slope = 1; dotted line). Piglet losses due to predation are likely evident in sows that were recorded as having few piglets born (rectangle; i.e., piglets were lost before they had been recorded), or for sows weaning far fewer piglets than were born (oval).

There were no statistically significant effects of parity, paddock ID, distance to native vegetation, or position within the farm on the weaning rate (Table 1). However, the weaning rate was significantly affected by sow ID (*F*_455,936_ = 1.28, *p* = 0.001): some sows were recorded as having very few piglets at farrowing, while others had a very low weaning rate (Figure 6).

### 3.3. Preliminary Trials to Test Bait Presentation and Placement

Presentation of baits: Cameras showed ravens regularly visiting bait stations, in addition to foxes, feral cats, and piglets. Of the 32 baits where we tested the four deployment methods, most baits were taken without triggering the camera and we could only confirm the identity of the animal taking a bait five times. Ravens were identified taking two surface-laid and two kangaroo hide-wrapped baits soon after they were distributed. A fox was observed digging up a buried bait more than a month after it was placed out.

Where to lay baits: Foxes visited 10 of the 36 surface-laid vs. buried bait stations that were monitored for one night (Table 2). Eight of these visits were to stations in open areas, possibly indicating that foxes are regularly using roads, and placing baits in open areas, making them easy for foxes to find. Foxes appeared to be more interested in the buried bait sites, while ravens generally do not dig up buried baits. Of the 36 baits placed out for one night, only one bait (surface-laid in vegetation) was taken by a fox. Ravens took six of the 18 surface-laid baits.

Tracking predator movements: Of the 32 piglet carcasses fitted with the GPS/VHF trackers, 12 were removed by foxes. Foxes moved transmitters an average of 171 ± 243 m (range 2–724 m) away from the original location. GPS tracking of piglet carcasses showed that foxes moved within native vegetation remnants, but also visited off-farm to neighboring paddocks, as well as taking carcasses across the farm away from the farrowing paddocks. Ravens were observed scavenging around all piglet carcasses and white-bellied sea eagles (*Haliaeetus leucogaster*) were observed scavenging carcasses in three instances.

## 4. Discussion

### 4.1. Identify the Level of Threat

Fox predation was identified as a major issue on the pork operation examined, and is likely to contribute to a significant reduction in the number of piglets weaned across many similar outdoor piggeries in Australia. We suggest that the impact of fox predation in outdoor piggeries has been significantly underestimated, because foxes take some newborn piglets born overnight before they are even recorded by piggery staff. At present, confirmed fox predation is only recorded for carcasses that are obviously “chewed” upon, but piglet carcasses monitored by camera or through GPS trackers revealed that foxes removed the carcasses from paddocks to eat them (possibly under cover). Therefore, far fewer losses are attributed to foxes than are likely to be occurring.

**Figure 7:**
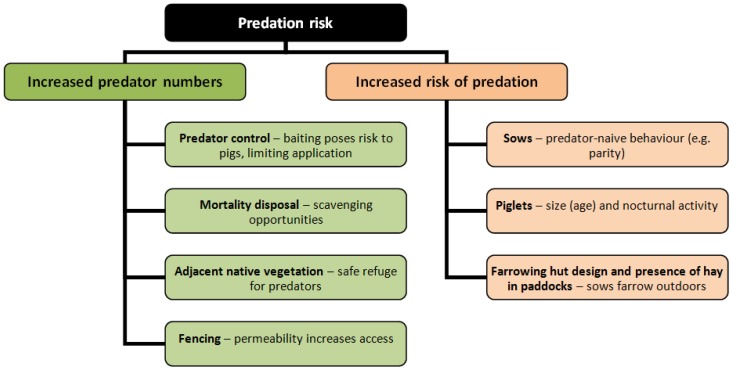
Summary of factors that lead to increased numbers of predators (e.g., red foxes) on outdoor piggeries and increased risk of fox predation.

Many carnivore species readily exploit high-energy anthropogenic food sources, leading to subsidized predator populations, e.g., [16]. Having easy food sources will therefore contribute to increased fox population size, which would increase vulnerability of small piglets (Figure 7). We had evidence of predator subsidization at this operation, where we recorded the presence of unfit animals (e.g., three-legged and mange-infested animals) that had full stomachs and therefore were apparently well-fed. The presence of easy food items (e.g., scavenging from the mortalities pit, stillborn carcasses, afterbirth, undefended young) would sustain large numbers of foxes, and although it was not possible to estimate the fox population size at this pork operation due to lack of individual markings for most animals, the presence of five individuals on a single camera image is a stark indication of a thriving population. It is possible that if foxes do not have access to carcasses through appropriate control of access to the carcass pit, then the existing large predator population may switch to taking live piglets. This emphasizes the need for coordinated fox control alongside other management actions.

Ravens were identified by the piggery staff as the cause of piglet predation in a small number of instances. We observed large groups of ravens feeding on the piglet carcasses fitted with GPS trackers and also scavenging within the carcass pit. We also observed ravens on camera harassing piglets and grabbing them by the tail. Ravens or foxes were implicated in 18% (75 of 412) of piglet deaths in European wild boars kept in external enclosures [17]. Ravens are known to scavenge dead lambs (*Ovis aries*) and placentas, but have also been observed predating a small number of healthy, as well as sick, and dying lambs [18].

Australian White Ibis (*Threskiornis moluccus*) were observed in large flocks within pig paddocks. This species is known to be a pest in urban areas [19]. Farm workers anecdotally reported occasional instances of ibis killing piglets by pecking at umbilical cords, but no ibis predation events were observed in the current survey or on camera. Ibis are known to occasionally eat rats and mice [20] and a closely related species, the African Sacred Ibis (*Threskiornis aethiopicus*) will predate eggs and chicks [21]. The presence of these birds in large numbers on the farm also poses a potential risk as they are hosts of diseases of concern to pig farmers, including *Salmonella* spp., *Toxoplasma gondii* and *Leptospira* spp. [22].

Native raptors could potentially predate free-range piglets, but it is unlikely that they take a significant number of animals and may be more likely to opportunistically feed on carrion, as has been shown for lambs [23,24]. White-bellied sea eagles were observed scavenging three piglet carcasses fitted with GPS units, and white-bellied sea eagles have been reported to take dogs, cats, poultry, and possums in the Solomon Islands [25]. Although not observed in this study, Wedge-tailed eagles (*Aquila audax*) will feed on lamb carrion, but are known to occasionally kill lambs [23] and could potentially also take piglets.

### 4.2. Farrowing and Weaning Records—Factors that Increase Risk of Fox Predation

Piglets only appeared to venture beyond the farrowing paddock boundaries during daytime (Figure 4d), and therefore should not be more vulnerable to predator predation during the day. However foxes did not limit their activities temporally, showing activity during the day (Figure 4a–c). Foxes also moved across the farrowing paddocks, taking piglets in front of farrowing huts (Figure 3). Piglets were therefore vulnerable even when they remained close to their sow.

Newborn and younger piglets appear to be the most vulnerable to predation, with more small piglets recorded as “missing”, as well as photographic evidence of young animals being taken by foxes. When left alone outside the farrowing hut at night, piglets showed limited awareness of the threat posed by a fox, even when it was less than 1 m away (Figure 3). Anecdotal records (piggery staff observations) suggested that piglet losses were especially marked for sows farrowing out in the paddock, where the sow would be distracted and unable to defend her young. However, even older piglets are also lost; for example, we observed a sow feeding her 2–3-week-old piglets outside the farrowing hut at night, and even when the sow was present, she was approached by a fox that attempted to take a piglet. Older piglets would also be increasingly vulnerable if they roamed farther from the farrowing paddocks.

We found no significant effect of distance from native vegetation on the weaning rate. However, camera trap data and the GPS tracking of carcasses demonstrated that foxes were using native vegetation remnants within the operation and in surrounding properties as refuges and as places to take food (piglet carcasses) to consume. Foxes were active in the middle of the day and far from protective cover, suggesting that there would be no greater protection for piglets born in paddocks that were located farther from native vegetation.

The standard practice of fostering piglets to sows with fewer young reduced our ability to use weaning and farrowing data alone to isolate the potential effects of predation on the weaning rate. Nevertheless, we found a significant effect of sow ID on the weaning rate (but no significant effect of sow parity). We captured photographic evidence of fox predation on newborn piglets from a farrowing hut camera, as well as fox predation of piglets that were suckling on the sow in front of farrowing huts, without the sows showing any obvious responses. It might therefore be that some sows lacked appropriate protective responses. Testing the behavioural responses of individual sows would be valuable to identify whether there is specific behaviour of individual sows that accounts for the effect of sow ID on the weaning rate.

### 4.3. Preliminary Trials to Test Bait Presentation and Placement

A questionnaire survey of outdoor pig farmers in Denmark indicated that 56% (of 153 farms) of farms regularly observed foxes, although only 43% (of 139 farms) applied permanent control measures [12]. The authors noted positive correlations between fox numbers and rodents on-farm, and the role of the predators as biological control agents was considered [12]. Although foxes may perform some level of beneficial rodent control, the abundance of foxes we recorded would clearly have a negative effect on piglet production numbers. Control measures are therefore warranted.

Cameras placed around the farm showed an abundance of fox activity. Despite a cull of nine foxes by the farm manager during the period we were monitoring (5 December 2014), there was no noticeable effect on the rate of fox capture events on camera. Intermittent fox shoots may only offer a short-term solution to reduce fox numbers [26], particularly where there is not a coordinated control effort across neighboring properties [27,28]. Additionally, because night spotlight shooting often relies on the ability of the hunter to “lure inquisitive and inexperienced animals into shooting range by rabbit whistle or, alternatively, to approach animals without them retreating” [29] (p. 227), foxes may become harder to detect and more likely to hide once they have experienced being shot at. Additional control measures are therefore required.

By the time we had located the GPS trackers attached to piglet carcasses, they had either slipped off or were chewed off the carcass. We therefore did not succeed in identifying the location of den sites using the monitored carcasses. However, the method could be improved to provide this ability in the future, to allow targeted den fumigation and destruction. Using GPS tracking collars on foxes would allow movements of foxes to be monitored to reveal core habitat resources for predators that could be targeted for control methods. The fate of tagged animals following different control methods could also be used as a measure of efficacy (mortality rates). These collars are currently AU ~$2000–$3000 each, but the technology is progressively becoming less expensive.

Because intensive methods for controlling foxes (e.g., shooting, den fumigation, and trapping) are labour intensive and costly, they consequently tend to be restricted to small areas [30]; the most commonly used fox control technique is lethal baiting [31]. There are opportunities for target-specific baiting, as foxes were frequently using patches of native vegetation across the farm and on neighboring properties. Based on GPS tracking, foxes tended to remain in the patches of native vegetation on the property. As piglets were less likely to move beyond the edges of these forest remnants, this result supports the current practice of targeting baiting within patches of native vegetation.

A concern with regard to toxic baiting would be movement of these baits by birds (reviewed by [31]), which could move baits into areas where they can be accessed by piglets. Buried, tethered or suspended baits may be suitable alternatives. Previous studies have shown that buried baits have the lowest non-target uptake compared with surface-laid baits [32,33,34], and we showed evidence that foxes were indeed interested in buried baits in this study. A fox was observed digging up a buried bait more than a month after it was placed out; if this had been a toxic (sodium fluoroacetate; “1080”) bait, it is unlikely it would still contain a lethal dose of 1080 to kill a fox due to leaching into the soil with rainfall or decay of 1080 by microorganisms [35]. Investigating different options that have increased longevity (e.g., poisoned chicken egg baits) may therefore be required.

Our preliminary study of bait-take showed only very few baits taken by foxes (one eighth buried bait after 1 month; 1/36 surface vs. buried baits monitored over one night) despite the cameras recording numerous “visits” to the vicinity of the bait. Foxes are particularly neophobic, and it is likely that our short monitoring period (a single night for the surface laid vs. buried baits) limited the bait take by foxes. This study should be repeated over a longer period to support these findings and also identify potential seasonal differences in bait-take.

### 4.4. Other Management Options to Reduce Predation Losses

We identified a number of factors that are likely to put piglets at greater risk of predation on the farm we worked at; some of these issues may be broadly relevant to other producers. These specific issues could be mitigated to some degree by management actions:

#### 4.4.1. Sows Farrowing in Paddocks at Night and Sows Feeding Piglets outside Farrowing Huts at Night

Outdoor pork operations provide shelters and nesting material for the sows, but hot Australian summer temperatures will often lure sows into farrowing outside, where piglets are more vulnerable to predation. Monitoring the activity of sows in parallel with the ambient temperature could indicate whether offering them alternative nesting sites (that have some measure of predator protection) can serve to reduce paddock farrowings. Gilts may be less likely to farrow in paddocks rather than huts if they are acclimatized to the huts for a few weeks before farrowing. Additionally, dispersing hay once farrowing huts have been moved as part of paddock rotation management could discourage sows from settling in open areas and even farrowing in the paddock at night.

#### 4.4.2. Carcass Pit

Dead pigs need to be immediately removed from the access of other pigs, with disposal occurring within 24 hours of death. National Environmental Guidelines for rotational outdoor pork operations [36] indicate that composting and rendering are the preferred methods for disposal of mortalities, stillborn piglets, and afterbirth from an environmental perspective, although suitable alternatives may include incineration and burial (subject to state/territory and government regulations). However burial is only recommended where rendering or composting are not feasible, with scavenging recognized as an issue [36]. The carcass pit clearly provided substantial food resources for foxes at the piggery under study. This could be reduced with predator-proof fencing, which requires a skirt (buried into the ground), a floppy top, and a few strands of electric wire; this costs up to $10,000 per km [37]. Also, daily application of lime would speed up decomposition and also reduce palatability of carcasses.

#### 4.4.3. Property and Paddock Fencing

Because the Australian system of rotational outdoor pork farming alternates between a pig phase and a crop/forage/pasture phase, most operators use electric fencing that is readily movable, allows for a flexible layout, and does not interfere with machinery movements during the crop/forage pasture phase [36]. The costs of predator-proof fencing would be prohibitive for internal paddock fencing, but may prove advantageous for property perimeter fencing.

#### 4.4.4. Guardian Animals

Recently, a number of studies have used guardian animals to successfully reduce livestock losses attributed to foxes. The presence of alpacas in a sheep flock increased the weaning rate of lambs by 12.83% compared to unguarded flocks [38]. Maremma sheepdogs (*Canis lupus familiaris*) have been successfully used long-term to protect young angora goats (*Capra aegagrus hircus*) from fox predation [39]. Similarly, Anatolian Shepherd dogs eliminated all losses due to fox predation across 20,000 head of free-range chickens (*Gallus gallus domesticus*) and alleviated the issue of reduced egg production in stressed chickens following fox incursions [39]. Guardian animals may be of use in outdoor pork operations to discourage foxes around the farrowing paddocks and to make foxes more wary. This option does require additional time and money investment to properly train animals to guard piglets, while defensive sows could also pose a risk to the dogs.

## 5. Conclusions

Crushing (“lay-overs”) accounts for a high percentage of piglet mortality in outdoor pork operations. For example, data for the UK indicate that lay-overs are responsible for more than 45% of total deaths where carcasses are retrieved to perform a post mortem (reviewed by [40]). In the present study, 76% of recorded deaths were noted as lay-overs, and similar figures are recorded for outdoor organic operations [3,4], indicating that this is indeed an important cause of death. However the numbers of animals taken by predators, before they are even recorded, should not be ignored.

Although there were few piglets recorded as lost to fox predation (i.e., “chewed” carcasses) in the present study, it is highly likely that foxes are contributing substantially to the 20% of piglets that were reported as “missing”, in addition to losses that were never recorded. Similar observations have been made by other authors, who also identify that piglets may roam large distances from their home paddocks in outdoor pork operations, increasing their vulnerability to fox or other predation, with the bodies not being found to record pre-weaning deaths [11].

The outdoor piggery we worked at weans an average of 1000 piglets per week for 3500 sows, compared with 1350 piglets for a similar-sized intensive operation in the same company (and therefore management and genetics). This equates to a 35% reduction in the number of piglets weaned for the outdoor farm. Some of the losses are due to increased crushing and lay-overs for the outdoor farm, although it has been noted that with unsupervised farrowings in outdoor systems, it can be difficult to distinguish piglets born dead from those which die in the hours immediately following birth [10]. However, predation is also likely to be a significant cause of piglet losses, and control of predation on outdoor pork operations is therefore a significant issue.

Although predator control will require careful planning (e.g., fencing, farrowing hut design, mortalities management), and investment (e.g., fencing, control, guardian animals), the financial and welfare gains to be made are also substantial. For example, the 20% loss due to “missing” piglets could equate to $20,000 per week under current prices for weaner piglets (Cost of Production estimated around AUD 60–70 ea, and buyers would expect to pay a premium for outdoor-raised animals; totaling ~AUD 100 ea)—a reasonable sum to offset against the additional costs required for predator-proofing such a facility.

## Figures and Tables

**Table 1:** Mixed model ANOVA results indicate sow ID has a significant effect on the weaning rate (proportion of piglets weaned to born live).

		df	df		
Effect	Effect type	Effect	Error	*F*	*P*
Sow ID	Random	455	936	1.28	0.001
Parity	Fixed (covariate)	1	467	0.37	0.544
Paddock ID	Random	68	869	1.00	0.475
Distance to native vegetation	Fixed (covariate)	1	30	0.06	0.816
Position on farm	Fixed	2	30	2.38	0.110

**Table 2:** Fate of 36 baits placed in open vs. forest sites. Bait stations were monitored over 1 day.

		Open Sites		Forest Sites	
Species	Action	Buried	Surface-Laid	Buried	Surface-Laid
Fox	Visited	6	2	1	1
	Took bait	-	-	-	1
Raven	Took bait		3	2	3
Total baits deployed		9	9	9	9
